# Readiness of Polish Nurses for Prescribing and the Level of Professional Burnout

**DOI:** 10.3390/ijerph16010035

**Published:** 2018-12-24

**Authors:** Anna Bartosiewicz, Paweł Januszewicz

**Affiliations:** Institute of Nursing and Health Sciences, Faculty of Medicine, University of Rzeszów, 35-959 Rzeszów, Poland; pjanusz888@gmail.com

**Keywords:** occupational burnout, LBQ questionnaire, prescription, nurse

## Abstract

Those in the medical profession, due to close contact and the emotional commitment of caring for patients, are particularly vulnerable to the occurrence of a phenomenon known as occupational ‘burnout’. The presented work deals with the problem of burnout and its relationship with new tasks undertaken by nurses. The aim of the study was an analysis of the relationship between the level of professional burnout of the nurses examined and their readiness to take on new duties related to writing prescriptions. The study was conducted among primary health care (PHC) and outpatient specialist care (OSC) nurses. The author’s questionnaire and the standardized Link Burnout Questionnaire (LBQ) were used. The highest level of occupational burnout was related to psychophysical exhaustion (16.00 ± 6.21). Higher results of occupational burnout among the nurses surveyed were matched by the lower readiness of the nurses to administer medicines and write prescriptions.

## 1. Introduction

The medical profession is characterized by intense and close contact with other people as well as high emotional involvement. This situation may result in a dangerous phenomenon referred to as ‘burnout’ syndrome [1]. The issue of occupational burnout has been addressed in the literature since the 1970s [2]. Freudenberger was the first to describe the burnout syndrome in literature, describing it as a state of exhaustion caused by excessive demands [3]. Maslach made a major contribution to the study of burnout. She, together with Jackson, conducted detailed research among such professions as teachers, doctors, nurses, psychologists, therapists, social workers, and policemen [4,5]. According to Maslach and Jackson, professional burnout is a syndrome of emotional exhaustion, depersonalization, and a reduced sense of one’s own accomplishments that can occur in people who work with people, where they assume excessive involvement which is linked with a very strong emotional identification with work [6]. On the other hand Pines and Aronson present occupational burnout as a state of physical, emotional, and mental exhaustion that is caused by prolonged emotional involvement [5,7].

In social professions, where close interpersonal contact plays an important role, it is important to be involved with and respond to the difficult situations of people seeking help [8]. This strenuous situation can cause a phenomenon known as occupational burnout [4]. This applies to the nursing profession. Nurses, on the one hand, enjoy great social trust, and on the other hand are burdened with high responsibility for the tasks performed [9]. High involvement and care for the well-being of patients are often the cause of the phenomenon of burnout among nurses [10,11]. According to researchers dealing with the issue of burnout, nurses belong to a professional group which, due to close contact with human suffering, often-difficult working conditions, and low pay, is particularly vulnerable to occupational stress. This can in turn can lead to the burnout syndrome [12,13]. The nursing profession, from the beginning of research on occupational burnout, was at the center of researchers’ interest [14]. The nurse with the right to practice is a competent professional, licensed to make independent decisions and organize nursing care in the health care system [2,15]. Professionalization of the profession is not only an honor, but above all involves a huge responsibility for the activities performed and ever-growing requirements to have an appropriate level of knowledge and competence. The new competences awarded to nurses in Poland from 1 January 2016, allowing them to write prescriptions, are a great breakthrough in Polish nursing, raising the status and importance of this profession [16,17]. Nurses with a Master’s degree and specializations in the field of nursing have the right to prescribe the medicines, foodstuffs, and medical devices listed on the list of the Ministry of Health. Nurses with undergraduate education may write prescriptions for medication and funds only as a continuation of treatment previously ordered by a doctor. Both groups of nurses must complete a specialist course on prescribing medicines [16,18]. In Poland, these solutions are a novelty but this is not so in other countries around the world. A pioneer in giving nurses such rights was the United States, followed by subsequent nations expanding nurses’ rights to write prescriptions. The scope of these rights varies depending on the distribution of the population, the health care system, and the professional status of nurses in a given country. However, the truth is that it brings many benefits to both patients and the entire health care system [19,20]. Miles et al. point out that strong policy and support for academic educational programs and nursing as a profession are key elements to effective development of the discussed issue [21]. The widespread practice of prescribing medicines by nurses varies from country to country. In Great Britain, it depends on the category of powers the nurse has, so nurses with independent powers can prescribe drugs from the full British National Formulary in all therapeutic indications in their area of competence. In turn, in countries struggling with underfunded health care and the problem of rapidly spreading diseases (AIDS/HIV) such as Malawi, Tanzania, Ethiopia, or Zambia, nurses play an important role in prescribing antiretroviral drugs. What is more, nurses in Uganda prescribe morphine because of the large number of people suffering from cancer. Dutch nurses have the power to prescribe medicines from their area of specialization. The demographic changes of the Dutch society and the large number of people with diabetes mean that nurses are very much involved in the care and prescription of medicines for these patients. [19,22]. Undertaking new tasks, however, requires the nurses’ commitment and effort, and in the case of certain illnesses, this can be extremely difficult and can undoubtedly lead to burnout syndrome.

The aim of the study was an analysis of the relationship between the level of professional burnout of the nurses examined and their readiness to take on new rights related to writing prescriptions.

## 2. Method

The research was conducted in the first half of 2016, among the primary health care (PHC) and outpatient specialist care (OSC) nurses in the south-eastern part of Poland in 13 randomly chosen treatment entities after obtaining consent from their managers. Before the proper study, a pilot study was carried out among 34 nurses to check understanding and correct completion of the questionnaire. The author’s questionnaire and the Link Burnout Questionnaire (LBQ) were used. All scale questions correlated positively with each other, as well as with the overall scale score. The obtained result indicates a good understanding of the tools. 

The study participation was voluntary and anonymous. The respondents first received oral information about the study and then written information about its purpose, voluntary nature. The respondents were assured that their consent or refusal to participate would not affect their continued employment in a given health care institution. To ensure data confidentiality, questionnaires were marked with numbers. Correctly completed questionnaires were equivalent to the consent of the participants to participate in the study. Consent was expressed by signing a consent form attached to the questionnaire. The questionnaires were provided to the respondents directly during face-to-face meetings. The purpose and validity of the study was also explained, while the nurses involved in the study obtained information about how to correctly complete the form. Overall, 1300 questionnaires were distributed, of which 800 (i.e., 60%) were collected back. After verifying all the questionnaires, 44 questionnaires were incompletely completed and for this reason were rejected. Data from the remaining 756 surveys were analyzed using statistical methods. In addition, the examined nurses (756) were a representative sample (25%) of all PHC (1923) and OSC (1102) nurses employed in this region of Poland. 

The research method used in this work was a diagnostic survey carried out using a questionnaire technique. The author’s survey questionnaire was composed of two parts. The first part of the questionnaire contained questions about sociodemographic variables of the subjects. The second part of the questionnaire contained questions about the opinions and knowledge of the respondents regarding the prescribing of medicines and writing prescriptions. In order to examine readiness to prescribe prescriptions, two components were distinguished, which sum up 69.2% of the variance in total (the first explains 48.7% of the variance and the other 20.5% of the variance—together they give a satisfactory result of almost 70% of the explained variance). In the first component we have foodstuffs for nutritional use, medical devices, and medicines ordered by a doctor, and in the other component, powerful drugs, intoxicants, psychotropic drugs, and antibiotics. The questionnaire was designed based on the five-point Likert scale.

In addition, the Link Burnout Questionnaire (LBQ) was used, which is the Polish adaptation of the Italian Link Burnout Questionnaire by Massimo Santinello (2008) [23]. The publisher is the Psychological Tests Laboratory of the Polish Psychological Association in Warsaw (year of issue 2014) [23]. The LBQ is designed to measure burnout in people whose work is related to helping other people and teaching. The questionnaire consists of 24 statements, divided into four subscales and allowing the assessment of occupational burnout in four aspects:

- Psychophysical exhaustion. This is the dimension concerning the assessment of one’s psychophysical resources. One end shows the degree of exhaustion, fatigue, tension, and work under pressure, and the other end shows the feeling of being active and full of energy. The person who gets higher results feels tired, has the feeling that he cannot cope with his duties anymore, and does not see the possibility to regenerate his strength.
- Lack of commitment to professional relationships. This dimension describes the quality of customer/patient relationships. At one end, clients/patients are treated incorrectly, from a distance and even in a hostile way by them, while at the other end there is involvement and individual cordial treatment for each client/patient. A person achieving high results in this area tends to treat clients/patients in a way that is cynical and even hostile.
- Feelings of lack of professional effectiveness. This refers to the assessment of one’s own professional competences. One end of this dimension is characterized by a sense of efficiency at work, efficiency in achieving professional goals, while the other involves a sense of inefficiency and no results at work. A person with higher scores in this dimension has a sense of inadequate self-efficacy and a sense of professional failure.
- Disappointment. Existential expectations, related to the specific motivation of people choosing a profession related to helping others. These people treat their profession as a mission, but contact with the professional reality (monotony, lack of cooperation, hardly visible work results) often leads to disappointment. At one end of this dimension, there is a passion, job satisfaction, and enthusiasm, while at the other end there is disappointment and a lack of enthusiasm. A high score person feels deeply disappointed in their work, which in their opinion deviates from previous expectations. The person believes that work does not give them opportunities for personal and professional development. This dimension is most related to the lack of job satisfaction [23,24].

The results may range from 6 to 36 points. The obtained results should be referred to the norms and in this way we obtain the ranges expressed in sten scores:

Score of 1–3 sten (6–10 points): These are low results, indicating no symptoms of burnout in the subjects.

Score of 4–7 sten (11–25 points)—these are average results, indicating the possibility of occurrence of certain problems related to burnout

Score of 8–10 sten (26–36 points) —these are high scores, indicating a high level of occupational burnout.

Answers are given on a six-level adjective scale, the subsequent points refer to the frequency with which these feelings appear: never, rarely, once or more per month, more or less every week, several times a week, and every day. The LBQ is used in diagnosing employee teams, departments, and organizations, as well as for preventive purposes for monitoring burnout in groups as well as individuals. It is also used as a supplement to tools designed to assess occupational functioning [24]. The Cronbach’s alpha coefficient of internal compliance for LBQ scales is as follows: psychophysical exhaustion, 0.77; lack of commitment to customer relations, 0.70; sense of ineffectiveness, 0.63; disappointment, 0.84. Factor analysis for the LBQ (varimax rotation) explained 50.98 % of variance, which is a satisfactory result, comparable to the result obtained by the author of the questionnaire. 

Criteria for selecting respondents who agreed to participate in the study:

Inclusion criteria: Nurses working in PHC and OSC, registered with the District Council of Nursing, with the right to practice, with at least one year of professional experience, and who were willing to participate in the study. 

Exclusion criteria: Nurses working in facilities other than PHC and OSC, non-registered nurses without the right to practice, nurses who had less than one year of work experience, and other employees of the PHC and OSC. 

### 2.1. Statistical Methods

To present statistical data, the method of descriptive statistics was used, through the arithmetic mean (M), the value of which determines the average level of a given variable, and the standard deviation (SD), the statistical measure of the results spreading around the expected value.

Also used were independence tests (×2), taking into account tables 2 × 2, the Yates continuity correction, and the Fisher’s exact test results for verification of differences between variables measured on the qualitative scale.

In order to compare the proportions of columns between the studied groups of nurses, the Bonfferoni method was used.

The Mann-Whitney U test and Kruskal-Wallis test for calculating the Spearman rho correlation coefficient were used to check differences between quantitative variables. This was caused by the lack of normality of quantitative variable distributions (verified by the Kolmogorov–Smirnov and Shapiro-Wilk tests) and the lack of parallel tests.

The level of significance was *p* < 0.05.

The calculations were carried out with the IBM SPSS Statistics 20 program (IBM, Armonk, NY, USA). 

### 2.2. Ethics Approval and Consent to Participate 

The research project carried out in accordance with the Helsinki Declaration. The study was approved by Bioethics Committee at the University of Rzeszow on 2 December 2015, Resolution number 12/12/2015.

## 3. Results

### 3.1. Characteristics of the Study Group

The average age of the respondents was 47.76 ± 9.65 years and ranged from 22 to 69 years. Almost half of the respondents—343 (45.4%)—were nurses with secondary nursing education. The second group in terms of numbers was represented by nurses with the professional title of Bachelor of Nursing (higher vocational education) with 157 respondents (20.8%), and the third group was composed of participants with higher education, at 104 respondents (13.8%). A nursing education and specialization in nursing was shown by 73 (9.7%) people, while the first degree and nursing specialties were held by 38 (5.0%) nurses. Only 41 (5.4%) people had a second-level Master’s degree and a specialization in nursing. Over half of the respondents—411 (54.4%)—had worked in the profession of a nurse over 20 years. Many—159 (21.0%)—had been employed for 16–20 years. Less numerous groups of nurses had been employed in the profession for 1–5 years (47; 6.2%), from 6 to 10 years (68; 9.0%), and from 11 to 15 years (71; 9.4%). The most frequently mentioned health care facility in which nurses had basic employment were public health care institutions, with employment contracts (644; 85.2%). In non-public healthcare institutions, 112 (14.8%) of the respondents were contract workers. Just over half of nurses (404; 53.4%) had taken a specialist course, 345 (45.6%) had a qualification course in the field of nursing, and almost every third respondent had completed a training course (247; 32.7). There were 141 (18.7%) people specializing in nursing, and 59 (7.8%) nurses had other forms of postgraduate improvement (post-graduate studies).

### 3.2. Results of Own Research

The level of occupational burnout included four subscales, of which the results of each scale could be in the range of 6–36 points. Higher scores were matched by higher levels of occupational burnout. The highest level of occupational burnout was related to psychophysical exhaustion (16.00 ± 6.21). The results of this scale were in the range of 6–36 points, and half of the people obtained no more than 15 points. Another category related to professional burnout was disappointment (15.43 ± 6.68). To a slight degree, occupational burnout in nurses was manifested by lack of involvement in professional relationships (14.98 ± 5.30) and the feeling of lack of professional effectiveness (14.97 ± 3.57). Table 1.

The analysis of our own research showed that a significantly higher level of professional burnout associated with the feeling of lack of professional effectiveness concerned PHC nurses (15.22 ± 3.55) rather than nurses employed in OSC (14.66 ± 3.57). In the case of the remaining three subscales of occupational burnout, statistically significant differences were not found (Table 2).

In the case of psychophysical exhaustion, average scores predominated and applied to the majority of nurses surveyed (*n* = 528, i.e., 69.8%), and low scores were in the second place and were related to 20.8% of nurses (*n* = 157). The high level of burnout of nurses in this subscale concerned 9.4% of nurses (*n* = 71).

**Figure 1:**
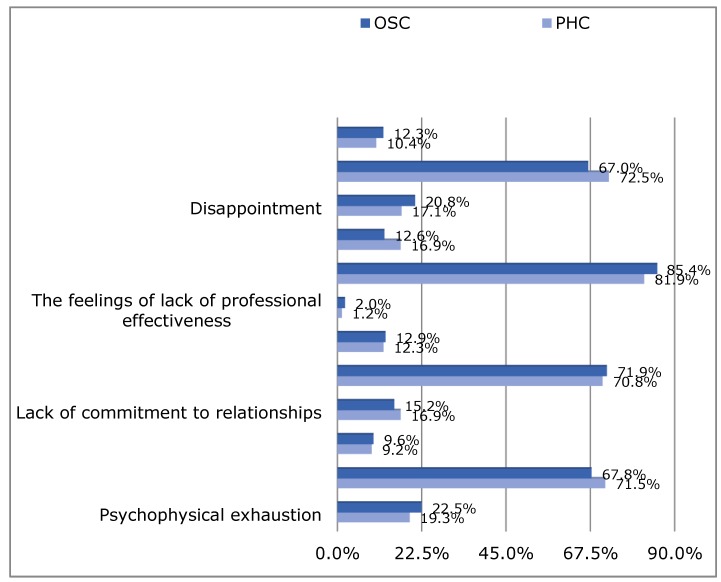
The level of occupational burnout (LBQ) according to the studied PHC and OSC groups of nurses.

The level of occupational burnout related to the lack of involvement in the professional relationships was at an average level and concerned a significant number of nurses surveyed (*n* = 539, i.e., 71.3%). Low results concerned 16.1% of nurses (*n* = 122), while high results of occupational burnout level concerned 12.6% of respondents (*n* = 95). The level of professional burnout associated with the sense of lack of professional effectiveness concerned 631 nurses (i.e., 83.5%) at the medium level. Low results in this area were obtained by 1.6% of respondents (*n* = 12), and high by 14.9% (*n* = 113). Most nurses (*n* = 529, i.e., 70.0%) obtained average results related to occupational burnout understood as disappointment. Low results in this scale were found in the group of 18.8% of people (*n* = 142), and high in the case of 11.2% of subjects (*n* = 85). The level of occupational burnout in individual subscales considered as categorical variables did not differ significantly between the PHC nurses and OSC nurses (Figure 1).

### 3.3. Socio-Demographic Factors and the Level of Occupational Burnout of the Examined Nurses.

The level of occupational burnout in the study group depended on selected sociodemographic factors of the nurses examined. Age affected the level of professional burnout: younger nurses experienced higher levels of occupational burnout and psychophysical exhaustion and lack of involvement in professional relationships than older nurses. Occupational burnout related to lack of involvement in professional relationships more often concerned nurses who did not have children or had one child. Having a Master’s degree resulted in an increase in the level of professional burnout associated with lack of engagement in professional relationships. With the increase in seniority, the level of lack of engagement in professional relationships decreased, while a higher sense of inefficiency in professional care concerned nurses working for 6–15 years, and disappointment concerned more often nurses working for more than 10 years. Higher levels of professional burnout were associated with lack of engagement in professional relationships and disappointment in nurses who did not have additional qualifications. In addition, the level of occupational burnout increased along with a deterioration in the material situation and self-esteem of health. The form of employment, i.e., lack of a permanent employment contract, contributed to an increase in the level of occupational burnout associated with psychophysical exhaustion, lack of engagement in professional relationships, and disappointment, as compared to people who had an employment contract for an indefinite period. Other factors, such as marital status, place of work, and residence, as well as the way of performing work for one or many principals had had little or no significant effect on the level of occupational burnout among the nurses surveyed (Table 3).

### 3.4. Level of Burnout and Nurses Readiness to Write Prescriptions

The studies showed that the level of professional burnout of nurses partly affected the readiness to prescribe medicines. To the greatest extent it reduced the readiness to prescribe medications previously commissioned by a doctor because in all areas of professional burnout the higher results corresponded to a lower willingness to prescribe these medications (rho < 0). It was also shown that occupational burnout significantly reduced the preparation of nurses for prescribing highly active drugs. The area of professional burnout associated with disappointment was the most important (rho = −0.131). Disappointment also had the greatest negative impact on readiness to prescribe medical devices (rho = −0.155). Correlations between the other criteria of occupational burnout and the preparation for prescribing selected drugs/measures were of little or no relevance at all (Table 4).

Among PHC nurses, professional burnout significantly reduced the readiness to prescribe especially medicines previously ordered by a doctor. All areas of occupational burnout negatively correlated with the preparation for prescribing this type of drugs. It was also shown that psychophysical exhaustion significantly reduced the readiness to prescribe strong drugs (rho = −0.143) and medical devices (rho = −0.107). Lack of involvement in the professional relationship with the patient negatively affected the readiness of the PHC nurses to prescribe medical devices (rho = −0.130) and foodstuffs for special nutritional purposes (rho = −0.119). Occupational burnout associated with disappointment significantly reduced the preparation of nurses for prescribing foodstuffs for particular nutritional uses (rho = −0.098), medical devices (rho = −0.115) and strong-acting drugs (rho = −0.124). Table 5.

Among OSC nurses, the impact of occupational burnout on the willingness to prescribe selected medicines was not significant. It was found that nurses with higher levels of occupational burnout associated with disappointment also had a lower level of preparation for prescribing highly active drugs (rho = −0.140). To a small extent, the lack of engagement in the professional relationship reduced the readiness to administer psychotropic drugs (rho = −0.113), the sense of lack of professional effectiveness reduced the preparation for prescribing highly active drugs (rho = −0.110), and disappointment reduced the readiness to prescribe medical devices (rho = −0.116). Table 6.

The analysis of the obtained results indicated how the readiness of nurses to write prescriptions in individual subscales of occupational burnout was developing.

The level of psychophysical exhaustion did not significantly affect the readiness of nurses to prescribe medicines. It was found, however, that between the PHC and OSC nurses with the average level of psychophysical exhaustion there were statistically significant differences in readiness for prescribing selected drugs. PHC nurses with an average level of psychophysical exhaustion had higher preparation for prescribing medical devices, narcotic drugs and psychotropic drugs than OSC nurses with an average level of psychophysical exhaustion (Table 7).

PHC nurses who had low levels of lack of commitment to professional relationships had a higher level of readiness to prescribe foodstuffs for particular nutritional uses than OSC nurses with low levels of occupational burnout in this dimension. Small differences were also found in groups of nurses with the average level of professional burnout associated with lack of involvement in relationships. PHC nurses who had an average lack of engagement in relationships had a higher readiness to prescribe narcotic drugs and psychotropic drugs than OSC nurses (Table 8).

The analysis of our own research results showed slight statistically significant differences between PHC nurses and OSC nurses with an average sense of lack of professional effectiveness in readiness for prescribing selected medicines. PHC nurses in this group had a higher level of preparation for prescribing foodstuffs for particular nutritional uses, medical devices, and psychotropic drugs than OSC nurses (Table 9).

Among PHC nurses with low level of occupational burnout associated with disappointment, readiness to administer medicines previously ordered by a doctor was significantly higher than in the same group of OSC nurses. It was also shown that PHC nurses with an average level of disappointment had greater preparation for prescribing narcotic drugs and psychotropic drugs than their colleagues working in OSC, with the same level of professional burnout associated with disappointment (Table 10).

## 4. Discussion

The issue of occupational burnout in relation to medical professions is a topic often raised in the literature [2,25,26]. The nurse’s work is multidimensional, and its specificity in terms of serving a sick or endangered person is often a source of satisfaction with respect to work and professional performance. However, intense and constant involvement in working with people and accompanying people in the disease is more likely to result in occupational burnout among this professional group [2,27]. Undertaking new tasks always requires a commitment and effort from a person, and in the case of certain ailments, new tasks may be too difficult to undertake. 

The aim of the study was to show the relationship between the level of professional burnout of PHC and OSC nurses and the readiness to prescribe medicines and write prescriptions. Higher results were matched by a higher degree of occupational burnout, which to the highest degree concerned psychophysical exhaustion, followed by disappointment and, to the least degree, lack of involvement and lack of professional effectiveness. According to Hagbaghery, the lack of self-confidence characteristic of occupational burnout may be an inhibitory factor in new entitlements, and vice versa, self-confidence helps in accepting new challenges [28]. Based on a literature review by Walkiewicz, et al. it can be seen that the most frequently used tool for measuring burnout was the Cristine Maslach questionnaire (the MBI (Maslach Burnout Inventory)) as well as the modified versions of this tool: the Human Services Survey (MBI-HSS) and the General Survey (MBI-GS) [29,30,31]. Due to the unavailability of a Polish version of the MBI questionnaire, the presented study was conducted using the new LBQ questionnaire [23,24]. 

The analysis of our own research showed that the significantly higher level of professional burnout associated with a sense of ineffectiveness concerned PHC nurses rather than nurses employed in OSC. The work of a PHC nurse in the patient’s environment and the related need to make independent decisions may be the reason for professional burnout more often than in the case of nurses working in other health care facilities. The analysis of the results of the conducted research—30 articles published over a 10-year period (from 2004 to 2014)—describing burn-out studies among Polish nurses reveals that Polish nurses are burned out to an average degree [30]. However, studies conducted by Dębska et al. among the PHC nurses showed a low level of occupational burnout, which may be related to a small number of nurses examined [32,33].

The study demonstrated that younger nurses experienced higher levels of occupational burnout associated with psychophysical exhaustion and lack of involvement in professional relationships than their older colleagues. This may be related to the fact that younger people exhibit greater professional activity, exploiting their strength and commitment excessively. This is confirmed by Jurado study though some studies indicate a lack of connection between age and occupational burnout. However, most researchers express the view that the level of occupational burnout increases with age [1,29,30,34,35]. In addition, the higher level of professional burnout associated with a lack of involvement in professional relationships occurred in nurses who do not have children or have one child. The results of other researchers in this respect are diverse: Wilczek-Rużyczka and Walkiewicz, referring to a study conducted by Eduardo des Santos et al., reported that a significantly higher predisposition to professional burnout was shown by nurses with a family than nurses that were single, which the author explains by the additional burden of household duties and emotional involvement in the affairs of their family [2,29]. This position also confirms the meta-analysis of 78 papers on the impact of sociodemographic factors on the occurrence of burnout syndrome [9]. Other examples from the literature also point to the relationship between seniority and the level of occupational burnout [29,36,37].

The factor that significantly influenced the level of professional burnout was also the level of education of the nurses surveyed. This is confirmed by Hallsten, who signaled the increased expectations, the fulfilment of more responsible functions, and the ensuing responsibility [38]. The above conclusions are not confirmed by Falby [39]. Different results have been obtained in relation to the possession of specialization by the nurses examined. People who did not have specialization in nursing had higher levels of professional burnout related to lack of engagement in the professional relationships, while nurses without additional qualification had higher levels of professional burnout associated with disappointment. According to Sowińska et al., a lack of access to professional training promotes burnout, which was also confirmed by the studies of Dębska et al. [40,41]. 

The cause of occupational burnout may also be an unsatisfactory financial situation and poor working conditions, as evidenced by the results of the conducted research. This is confirmed by other researchers, pointing to correlation between the most frequent complaints (headache, back and muscle pain, weakness, chronic fatigue, and irritability) and increased susceptibility to burnout syndrome [30,41]. However, the workplace was not a factor significantly increasing the level of occupational burnout, as confirmed by the results of the Dębska et al. which, however, cannot be used as basis due to the small number of respondents [30,41]. The results of other researchers indicate, in turn, a higher level of occupational burnout among nurses working in surgical wards, ophthalmology oncology, hematology, and intensive care units, with particular emphasis on the burn-out and workload of pediatric nurses [13,42,43]. The very low level of professional satisfaction among environmental nurses may indicate a high risk of burnout in this group [44,45].

The analysis of our own results regarding the correlation of occupational burnout between PHC and OSC nurses on the readiness to prescribe medicines and write prescriptions has shown that there is a partial relationship between the above mentioned factors. To the greatest extent it reduced the readiness to prescribe medications previously ordered by a doctor because in all areas of professional burnout the higher results corresponded to a lower willingness to prescribe these medications. It was also shown that occupational burnout significantly reduced the preparation of nurses to prescribing potent drugs. The scope and type of medicines that can be prescribed by nurses differ and depend on the level of competence and health condition of a given country. [19,46]. A very important role in the attribution of drugs with a strong analgesic effect is played by North American nurses working in emergency services as well as nurses working in poor African countries, who fulfil an important role in the prescription of antivirals and morphine to patients, especially in remote areas and with difficult access to a doctor. Similarly, in Ireland, registered practice nurses have the authority to prescribe psychotropic and narcotic drugs to their patients [19,20,46]. Such responsible tasks require not only an adequate amount of knowledge but also psychological resilience, which can be difficult to achieve in the case of work burnout. This is in accordance with Jaworowska, the author of the Polish version of the LBQ manual, who concluded that the discouraged person loses passion and enthusiasm to perform their work as well as to take on new tasks [24]. 

Among PHC nurses, professional burnout significantly reduced the readiness to prescribe, especially medicines previously ordered by a doctor and also strong drugs, medical devices, foodstuffs for special nutrition, and strong-acting drugs. The list of drugs approved for prescription by nurses in Poland is made up of 16 groups of drugs containing 33 active substances and this is relatively small compared to other countries, e.g., Sweden, where the list of medicines contains about 230 medicines [46]. A significant factor influencing professional burnout among PHC nurses, especially those working in the environment, is constant, with intensive involvement in the social and physical problems of their charges [1]. Constant concern for others can certainly cause little motivation to take on new initiatives. According to Sęk, in the case of people with occupational burnout one can observe different ways of withdrawing from professional activity, or performing activities not so much necessary as essential, without any chance to undertake new challenges [5].

In case of OSC nurses, the impact of occupational burnout on the willingness for prescribing selected medicines was not significant. It was found that nurses with higher levels of occupational burnout associated with disappointment also had a lower level of preparation for prescribing highly active drugs. This attitude of nurses working in other healing entities may be connected with consciousness and with a sense of responsibility for undertaking such important tasks as ordering medicines for patients, especially strong ones (most of them had a specialization). According to Dębska et al., postgraduate education has a positive effect on the prevention of occupational burnout [41]. PHC nurses with an average level of psychophysical exhaustion had higher preparation for prescribing medical devices, narcotic drugs and psychotropic drugs than OSC nurses with an average level of psychophysical exhaustion. The significant workload and the monotony of the activities performed can contribute to the development of burnout, and according to Ogińska and Żuralska, it can significantly affect the reduction of work efficiency and the willingness to undertake new tasks [47].

PHC nurses who have low levels of non-involvement in relationships had a higher level of readiness to prescribe foodstuffs for particular nutritional uses than OSC nurses with low levels of occupational burnout in this dimension. The analysis of our own research results showed slight statistically significant differences between PHC nurses and OSC nurses with an average sense of lack of professional effectiveness in readiness to prescribing selected medicines. Differences between PHC and OSC nurses may result from different conditions and requirements at work. The specificity and place of work differentiates nurses in terms of occupational burnout. Among PHC nurses where a low level of occupational burnout associated with disappointment, readiness to administer medicines previously ordered by a doctor was significantly higher than in the same group of OSC nurses. It was also shown that PHC nurses with an average level of disappointment had greater preparation for prescribing narcotic drugs and psychotropic drugs than their colleagues working in OSC, with the same level of professional burnout associated with disappointment.

Burnout syndrome is a multidimensional phenomenon whose consequences are visible in many spheres of human life: physical, mental, personal, and professional. The new competences awarded to nurses are undoubtedly a breakthrough in Polish nursing, providing an increase in occupational autonomy. These duties bring our nursing closer to other countries, where nurses take on some of the doctor’s qualifications and help in caring for a patient, becoming a member of an interdisciplinary team. [19,22]. Negative effects of professional burnout cause a minimalistic attitude. They are also an obstacle to the development of holistic function and to providing patient-oriented health care as a main goal. [2,4,6]. 

## 5. Conclusions

1. The level of occupational burnout among the nurses surveyed was moderate and concerned mainly psychophysical exhaustion and disappointment. The smallest degree of occupational burnout in nurses was manifested by a lack of engagement in professional relationships and by the feeling of lack of professional effectiveness.
2. A significantly higher level of professional burnout associated with the feelings of lack of professional effectiveness concerned PHC nurses rather than nurses employed in OSC. In the case of the remaining three subscales of occupational burnout, statistically significant differences were not found.
3. The level of professional burnout of nurses partly affected the readiness to prescribe medicines. To the greatest extent, it reduced the readiness to prescribe medications previously commissioned by a doctor because in all areas of professional burnout, a higher level meant a lower willingness to administer these drugs.
4. Occupational burnout significantly impaired the readiness of nurses for prescribing highly active drugs. The area of professional burnout associated with disappointment was the most important here. Disappointment also had the greatest negative impact on the readiness to prescribe medical devices.
5. The higher the level of occupational burnout among the nurses surveyed, the lower the readiness of the subjects to prescribe medicine and write prescriptions.

### 5.1. Limitations of the Study

There are limitations in the study. The first was the age of the respondents: the research was conducted in a group of nurses whose average age was 48 years. Secondly, the research was carried out in south-eastern Poland and, in the future, other regions of the country should also be included in the study. Thirdly, the study was carried out shortly after the introduction of the law authorizing prescription, and hence the nurses who took part in the study did not write prescriptions because they were in the process of qualifying for new entitlements. The new and unknown LBQ questionnaire may also be limitation of the study. 

### 5.2. Summary

These variables should be taken into account in the design of risk profiles for burnout in nursing professionals. This would help to implement prevention programs, such as nurses’ support groups for nurses who are most at risk of developing burnout. In this way, some of its more serious consequences could be avoided. Also, nurses themselves must take care to avoid occupational burnout by taking care of proper relationships at work, and by receiving proper rest and personal professional development. 

## Figures and Tables

**Table 1:** The level of occupational burnout of the nurses examined using the Link Burnout Questionnaire (LBQ).

LBQ		Psychophysical Exhaustion	Lack of Commitment to Professional Relationships	The Feelings of Lack of Professional Effectiveness	Disappointment
M		16.00	14.98	14.97	15.43
SD		6.21	5.30	3.57	6.68
Minimum		6.00	6.00	7.00	6.00
Maximum		36.00	35.00	29.00	34.00
Percentiles	Q1	11.00	11.00	13.00	10.00
	Q2 (Me)	15.00	14.00	14.00	15.00
	Q3	20.00	18.00	17.00	20.00

Q1: quartile I; Q2 (Me): quartile II (median); Q3: quartile III; M: arithmetic mean; SD: standard deviation.

**Table 2:** The level of occupational burnout (LBQ) according to the studied PHC and OSC group of nurses. PHC: primary health care; OSC: outpatient specialist care.

Examined Group		Psychophysical Exhaustion	Lack of Commitment to Professional Relationships	The Feelings of lack of Professional Effectiveness	Disappointment
PHC	M	15.95	14.83	15.22	15.42
	SD	6.12	5.39	3.55	6.51
OSC	M	16.06	15.16	14.66	15.44
	SD	6.32	5.20	3.57	6.88
Total	M	16.00	14.98	14.97	15.43
	SD	6.21	5.30	3.57	6.68
*p*		0.9299	0.2901	0.0193	0.9206

**Table 3:** Socio-demographic factors significantly influenced at the level of occupational burnout of the examined nurses.

Socio-demografic Factors	Psychophysical Exhaustion	Lack of Commitment to Professional Relationships	The Feelings of Lack of Professional Effectiveness	Disappointment
Age	0.0019	<0.0001	0.9546	0.5446
Level of education	0.3286	0.0091	0.6458	0.0652
Work experience	0.0003	0.0012	0.0098	0.0049
Additional qualifications	0.0151	0.0195	0.1623	0.0031
Form of employment	0.0041	0.0027	0.6222	0.0029
Material status	0.0005	0.0321	0.0328	0.4736
Health status	<0.0001	0.6773	0.0337	<0.0001

**Table 4:** The level of occupational burnout (LBQ) and readiness for administer medicines among all surveyed nurses.

Total (*n* = 756)		Psychophysical Exhaustion	Lack of Commitment to Professional Relationships	The Feelings of Lack of Professional Effectiveness	Disappointment
Foodstuffs for particular nutritional uses	rho	−0.025	−0.086	−0.044	−0.066
	*p*	0.4991	0.0182	0.2322	0.0684
Medical products	rho	−0.085	−0.107	−0.036	−0.115
	*p*	0.0189	0.0031	0.3229	0.0015
Potent drugs	rho	−0.115	−0.077	−0.090	−0.131
	*p*	0.0016	0.0344	0.0137	0.0003
Narcotic drugs	rho	−0.026	0.001	−0.049	−0.064
	*p*	0.4797	0.9718	0.1770	0.0781
Psychotropic medicines	rho	−0.046	−0.059	−0.063	−0.081
	*p*	0.2099	0.1054	0.0830	0.0263
Antibiotics, chemotherapy	rho	−0.034	−0.001	−0.036	−0.067
	*p*	0.3538	0.9840	0.3273	0.0655
Only drugs previously ordered by a doctor	rho	−0.079	−0.166	−0.099	−0.124
	*p*	0.0295	<0.0001	0.0064	0.0006

**Table 5:** Readiness for prescribing of medicines and the level of occupational burnout among PHC nurses.

PHC (*n* = 414)		Psychophysical Exhaustion	Lack of Commitment to Professional Relationships	The Feelings of Lack of Professional Effectiveness	Disappointment
Foodstuffs for particular nutritional uses	rho	−0.063	−0.119	−0.053	−0.098
	*p*	0.1990	0.0153	0.2857	0.0458
Medical products	rho	−0.107	−0.130	−0.021	−0.115
	*p*	0.0302	0.0079	0.6685	0.0194
Potent drugs	rho	−0.143	−0.084	−0.082	−0.124
	*p*	0.0035	0.0870	0.0960	0.0113
Narcotic drugs	rho	−0.019	0.050	−0.023	−0.056
	*p*	0.6976	0.3058	0.6420	0.2540
Psychotropic medicines	rho	−0.069	−0.013	−0.058	−0.084
	*p*	0.1595	0.7920	0.2355	0.0896
Antibiotics, chemotherapy	rho	−0.062	0.014	−0.052	−0.041
	*p*	0.2094	0.7731	0.2947	0.4045
Only drugs previously ordered by a doctor	rho	−0.162	−0.220	−0.179	−0.196
	*p*	0.0010	<0.0001	0.0003	0.0001

**Table 6:** Readiness for prescribing of medicines and the level of occupational burnout among OSC nurses.

OSC (*n* = 342)		Psychophysical Exhaustion	Lack of Commitment to Professional Relationships	The Feelings of Lack of Professional Effectiveness	Disappointment
Foodstuffs for particular nutritional uses	rho	0.024	−0.036	−0.053	−0.030
	*p*	0.6531	0.5034	0.3321	0.5786
Medical products	rho	−0.060	−0.073	−0.068	−0.116
	*p*	0.2662	0.1765	0.2110	0.0316
Potent drugs	rho	−0.079	−0.059	−0.110	−0.140
	p	0.1437	0.2729	0.0422	0.0094
Narcotic drugs	rho	−0.035	−0.057	−0.092	−0.074
	*p*	0.5160	0.2935	0.0901	0.1715
Psychotropic medicines	rho	−0.016	−0.113	−0.080	−0.078
	*p*	0.7624	0.0367	0.1391	0.1509
Antibiotics, chemotherapy	rho	−0.004	−0.013	−0.023	−0.094
	*p*	0.9456	0.8084	0.6711	0.0817
Only drugs previously ordered by a doctor	rho	0.017	−0.094	−0.015	−0.045
	*p*	0.7591	0.0811	0.7848	0.4102

**Table 7:** Readiness for prescribing of drugs and psychophysical exhaustion in PHC/OSC.

Psychophysical Exhaustion—PHC/OSC		Low		Average		High		*p*
		M	SD	M	SD	M	SD	
Foodstuffs for particular nutritional uses	PHC	2.78	1.38	2.75	1.36	2.55	1.43	0.6509
	OSC	2.44	1.42	2.54	1.32	2.48	1.15	0.7618
Medical products	PHC	3.34	1.51	3.51	1.38	2.97	1.42	0.0949
	OSC	3.27	1.51	3.26	1.43	3.00	1.30	0.4367
Potent drugs	PHC	2.25	1.28	2.03	1.17	1.71	1.01	0.0659
	OSC	2.09	1.23	1.92	1.08	1.79	1.02	0.4664
Narcotic drugs	PHC	1.51	0.75	1.57	0.86	1.47	0.69	0.8951
	OSC	1.60	0.89	1.42	0.77	1.55	0.94	0.2817
Psychotropic medicines	PHC	1.75	1.03	1.75	1.04	1.61	0.97	0.6518
	OSC	1.74	1.07	1.54	0.87	1.61	1.03	0.5389
Antibiotics, chemotherapy	PHC	1.95	1.25	1.74	1.03	1.74	0.95	0.5941
	OSC	1.57	0.92	1.74	1.00	1.67	1.05	0.3618
Only drugs previously ordered by a doctor	PHC	3.64	1.42	3.39	1.44	2.82	1.41	0.0121
	OSC	3.31	1.57	3.31	1.48	3.39	1.43	0.9693

**Table 8:** Readiness for prescribing of drugs and lack of commitment to relationships among PHC/OSC nurses.

Lack of Commitment to Professional Relationships—PHC/OSC		Low		Average		High		*p*
		M	SD	M	SD	M	SD	
Foodstuffs for particular nutritional uses	PHC	2.77	1.44	2.75	1.36	2.59	1.34	0.7483
	OSC	2.12	1.20	2.63	1.35	2.32	1.22	0.0254
Medical products	PHC	3.47	1.49	3.45	1.39	3.20	1.44	0.4578
	OSC	3.00	1.44	3.26	1.45	3.41	1.32	0.3814
Potent drugs	PHC	2.07	1.20	2.06	1.20	1.88	1.13	0.5440
	OSC	1.94	1.26	1.96	1.08	1.86	1.11	0.6474
Narcotic drugs	PHC	1.39	0.67	1.60	0.87	1.49	0.70	0.1702
	OSC	1.44	0.75	1.46	0.78	1.57	1.07	0.9881
Psychotropic medicines	PHC	1.61	0.94	1.80	1.08	1.59	0.83	0.3175
	OSC	1.69	1.00	1.58	0.91	1.57	1.00	0.6287
Antibiotics, chemotherapy	PHC	1.71	1.12	1.81	1.09	1.69	0.88	0.5161
	OSC	1.58	0.85	1.71	1.00	1.73	1.11	0.8701
Only drugs previously ordered by a doctor	PHC	3.64	1.55	3.38	1.41	3.02	1.45	0.0300
	OSC	3.58	1.49	3.27	1.49	3.30	1.47	0.3053

**Table 9:** Readiness for prescribing of drugs and the feelings of lack of professional effectiveness among PHC/OSC nurses.

The Feelings of Lack of Professional Effectiveness PHC/OSC		Low		Average		High		*p*
		M	SD	M	SD	M	SD	
Foodstuffs for particular nutritional uses	PHC	3.80	1.10	2.74	1.36	2.63	1.38	0.1751
	OSC	3.14	1.21	2.50	1.33	2.47	1.30	0.4078
Medical products	PHC	3.40	0.89	3.49	1.39	3.10	1.51	0.1226
	OSC	3.57	1.51	3.25	1.45	3.09	1.32	0.5103
Potent drugs	PHC	1.40	0.55	2.11	1.21	1.76	1.03	0.0399
	OSC	2.14	1.35	1.95	1.11	1.86	1.10	0.7327
Narcotic drugs	PHC	1.60	0.89	1.57	0.83	1.46	0.76	0.6000
	OSC	1.71	1.50	1.48	0.79	1.42	0.85	0.6458
Psychotropic medicines	PHC	1.60	0.89	1.78	1.05	1.56	0.93	0.1981
	OSC	1.29	0.49	1.61	0.95	1.51	0.88	0.6371
Antibiotics, chemotherapy	PHC	1.80	0.84	1.81	1.11	1.66	0.92	0.7203
	OSC	1.57	0.53	1.67	0.97	1.88	1.16	0.6515
Only drugs previously ordered by a doctor	PHC	4.40	0.55	3.47	1.42	2.89	1.51	0.0040
	OSC	3.43	1.51	3.36	1.49	3.07	1.49	0.4465

**Table 10:** Readiness for prescribing drugs and disappointment among PHC/OSC nurses.

Disappointment—PHC/OSC		Low		Average		High		*p*
		M	SD	M	SD	M	SD	
Foodstuffs for particular nutritional uses	PHC	2.90	1.30	2.72	1.36	2.56	1.48	0.3246
	OSC	2.52	1.39	2.54	1.31	2.33	1.26	0.6529
Medical products	PHC	3.62	1.40	3.41	1.41	3.21	1.49	0.2625
	OSC	3.49	1.54	3.20	1.38	3.02	1.51	0.1175
Potent drugs	PHC	2.21	1.33	2.05	1.15	1.72	1.12	0.0532
	OSC	2.11	1.33	1.94	1.02	1.69	1.14	0.0894
Narcotic drugs	PHC	1.52	0.81	1.60	0.85	1.26	0.54	0.0212
	OSC	1.48	0.73	1.45	0.77	1.60	1.15	0.8267
Psychotropic medicines	PHC	1.77	1.11	1.78	1.03	1.37	0.76	0.0202
	OSC	1.72	1.10	1.56	0.86	1.57	1.02	0.7673
Antibiotics, chemotherapy	PHC	1.92	1.31	1.78	1.03	1.58	0.93	0.3935
	OSC	1.69	0.98	1.71	0.98	1.60	1.11	0.3361
Only drugs previously ordered by a doctor	PHC	3.83	1.34	3.35	1.43	2.88	1.53	0.0020
	OSC	3.25	1.50	3.40	1.48	3.02	1.52	0.2911
